# Survival benefits from postoperative radiation therapy on lymph node positive patients with pancreatic adenocarcinoma

**DOI:** 10.18632/oncotarget.9620

**Published:** 2016-05-26

**Authors:** Zuguang Xia, Xiaoyan Jia, Kai Chen, Dapeng Li, Jing Xie, Hong Xu, Yixiang Mao

**Affiliations:** ^1^ Department of Medical Oncology, Fudan University Shanghai Cancer Center, Department of Oncology, Shanghai Medical College, Fudan University, Shanghai, China; ^2^ Department of Oncology, The First Affiliated Hospital of Soochow University, Suzhou, China; ^3^ Department of Gastrointestinal Medical Oncology, The University of Texas MD Anderson Cancer Center, Houston, Texas, USA

**Keywords:** pancreatic adenocarcinoma, postoperative radiation therapy, lymph node metastasis, survival

## Abstract

The benefit of combining postoperative radiation therapy (PORT) with chemotherapy for resected patients with pancreatic adenocarcinoma is controversial. We sought to determine the effects of PORT on survival in patients with pancreatic adenocarcinoma who underwent primary site surgery. Patients with pancreatic adenocarcinoma receiving primary tumor surgery between 1988 and 2012 were identified from the Surveillance, Epidemiology and End Results (SEER) database. We estimated the association between PORT and other clinicopathologic factors and survival. In total, 5304 patients were identified who underwent pancreatic resection including 2093 patients who had PORT and 3211 patients who had no PORT. Median overall, cancer-specific, and other-cause survival were 19.0, 20.0, and 196.0 months, respectively, with PORT versus 14.0, 15.0, and 163.0 months, respectively, without PORT (all *P* < 0.001). Subset analysis revealed that the benefit of PORT was limited to patients with N1 disease. Median overall, cancer-specific, and other-cause survival for patients with N1 disease were 18.0, 18.0, and NA months, respectively, with PORT versus 12.0, 13.0, and 154.0 months, respectively, without PORT (all *P* < 0.001). Regardless the number of positive lymph node count (PLN) and lymph node ratio (LNR), PORT was always associated with increased survival on multivariate analysis in patients with N1 disease (all *P* < 0.001). In summary, survival benefits might be obtained from PORT on lymph node positive patients with pancreatic adenocarcinoma.

## INTRODUCTION

In 2015, an estimated 48960 individuals were diagnosed with pancreatic cancer in the United States and approximately 40560 individuals died from the disease [1]. After adjusting for age, the numbers of new cases and deaths from pancreatic cancer were 12.4 and 10.9, respectively, per 100000 individuals per year between 2008 and 2012. The rate of new cases has been rising an average of 0.8% each year over the past decade. The 5-year survival rate for patients with all stages of pancreatic cancer is 7.2% [1].

Currently, surgery is the best treatment for early pancreatic cancer. However, only 10–35% of stage I patients will live more than five years after surgery. To help increase the chance of being cured, and improve survival of patients with early-stage cancer, a combination of adjuvant chemotherapy (ie. 5-fluouracil or gemcitabine) and/or radiation therapy is often given in addition to surgery. It has been suggested that subsets of patients with pancreatic cancer, such as patients with positive lymph nodes, may be more likely to benefit from adjuvant chemoradiation. However, studies have found mixed results. A meta-analysis of 4 randomized controlled trials found that adjuvant chemoradiation had a similar lack of benefit in lymph node-positive and -negative patients [2]. However, an exploratory subset analysis of 94 patients who underwent distal pancreatectomy at Johns Hopkins Hospital suggested that patients with positive lymph nodes derived greater benefit from adjuvant chemoradiation than those with negative nodes [3]. Previous analyses of the SEER database suggested an advantage for adjuvant radiation therapy after pancreatic tumor resection [4–6]. Subgroup analysis revealed that the benefit of PORT was limited to lymph node-positive (N1) patients [6]. Intense interest has centered on similar considerations in the addition of postoperative radiation therapy (PORT) to standard therapy such as chemotherapy and survival in pancreatic cancer. However, consensus on the efficacy of PORT has not been made. We therefore performed a retrospective study to evaluate the association of PORT and survival in patients with pancreatic adenocarcinoma receiving standard therapy.

## RESULTS

### Patient characteristics

We identified 5646 patients who had undergone tumor surgery for a first primary pancreatic adenocarcinoma diagnosed between 1988 and 2012. We excluded 154 patients without accurate total lymph node count (TLN) information and 1 patient without positive lymph node count (PLN) information. The remaining 5491 patients included 5178 who underwent lymph node resection and 313 who did not. A total of 5304 patients were included in the final analyses: 2093 patients who had undergone PORT and 3211 patients who did not receive treatment with radiation (187 patients treated with preoperative radiation therapy were excluded). The median age at diagnosis was 65 years (interquartile range, 58–73 years). The overall median follow-up time was 56 months. Compared with patients who had not undergone PORT, those who had undergone PORT were more likely to be younger, to have regional-stage disease, to have disease located at the head of the pancreas, and to have well- or moderately well-differentiated tumors (Table 1).

**Table 1:** Patient and tumor characteristics and outcomes (*n = 5304*)

	No PORT *N* (%)		PORT *N* (%)		*P* value
**Number of patients**	3211		2093		
**Year of diagnosis, median (range)**	2007 (1988–2012)		2006 (1988–2012)		
**Age, median (interquartile range)**	67 (59–75)		63 (56–70)		< 0.05 (χ^2^ test)
**Sex**					0.312
Male	1599	(49.8)	1072	(51.2)	
Female	1612	(50.2)	1021	(48.8)	
**Race or ethnicity**					0.038
White	2638	(82.2)	1679	(80.2)	
Black	286	(8.9)	231	(11.0)	
Others	287	(8.9)	183	(8.7)	
**Cancer stage**					< 0.001
Localized	451	(14.0)	210	(10.0)	
Regional	2167	(67.5)	1695	(81.0)	
Distant	581	(18.1)	181	(8.6)	
Unstaged	12	(0.4)	7	(0.3)	
**Tumor location**					< 0.001
Head of pancreas	2239	(69.7)	1559	(74.5)	
Body or tail of pancreas	561	(17.5)	330	(15.8)	
Other	411	(12.8)	204	(9.7)	
**Tumor differentiation**					
Poorly differentiated or undifferentiated	1111	(34.6)	652	(31.2)	0.001
Well or moderately differentiated	1828	(56.9)	1294	(61.8)	
Unknown	272	(8.5)	147	(7.0)	
**Total number of LN, median (range)**	12	(0–69)	12	(0–72)	
**Number of PLN, median (range)**	1	(0–32)	1	(0–34)	
**LNR (mean ± SD)**	0.18 ± 0.23		0.17 ± 0.22		
**Lymph node status**					< 0.001
Not examined	231	(7.2)	74	(3.5)	
pN0	1118	(34.8)	699	(33.4)	
pN1	1862	(58.0)	1320	(63.1)	
**Median overall survival, months 95% CI**	14.0 (13.3–14.7)		19.0 (18.2–19.8)		< 0.001
**Cancer-specific survival, months 95% CI**	15.0 (14.2–15.8)		20.0 (19.1–20.9)		< 0.001
**Other-cause survival, months 95% CI**	163.0 (129.5–196.5)		196.0 (160.2–231.8)		< 0.001

Abbreviations: PORT, postoperative radiation therapy; LN, lymph nodes; PLN, total number of positive lymph nodes; LNR, lymph node ratio; CI, confidence interval.

# Mann-Whitney *U* test.

### Lymph node status and survival

**Figure 1:**
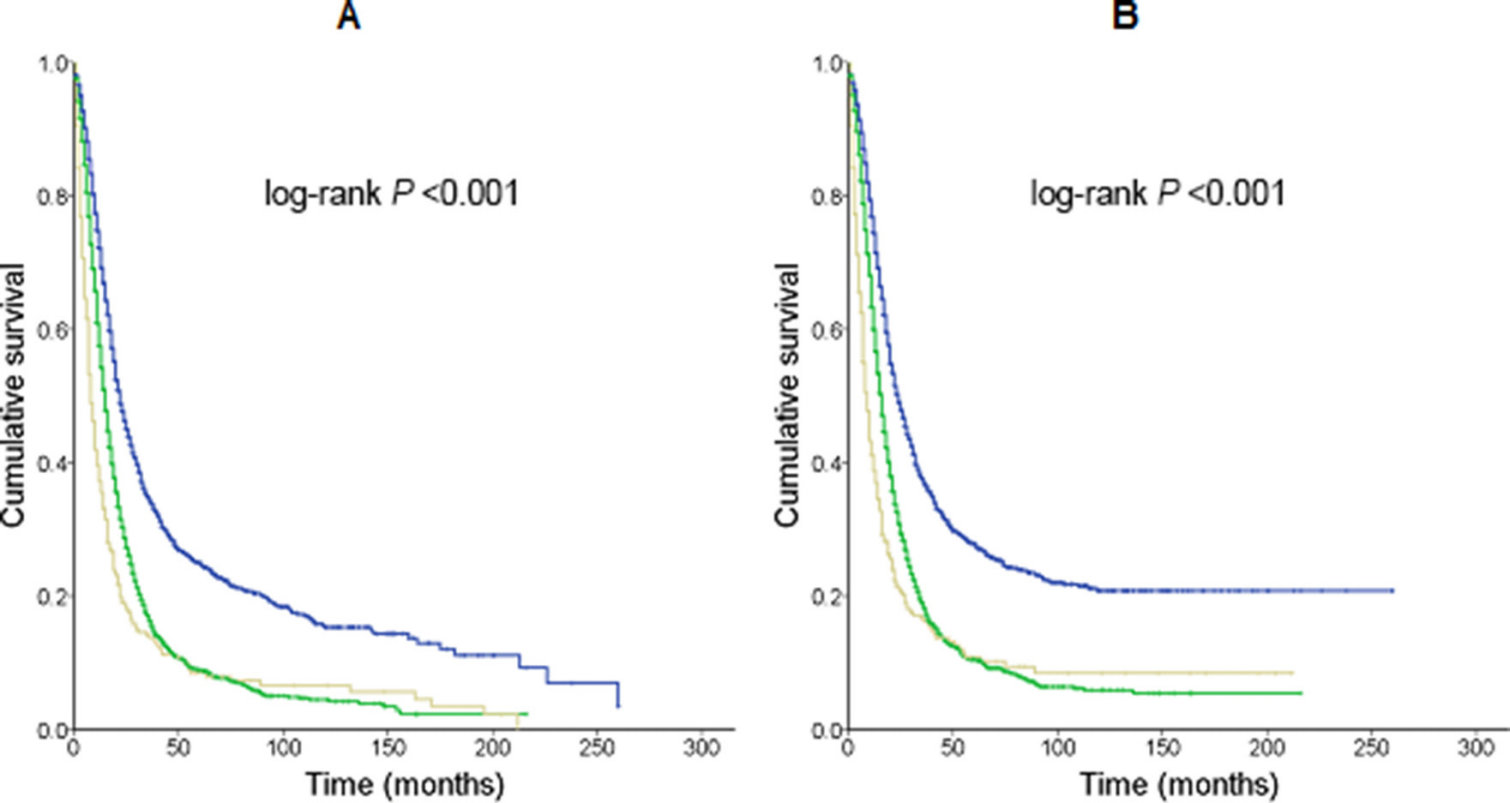
Kaplan-Meier survival curves for patients who had undergone primary tumor resection stratified by total and positive lymph node numbers. Median survival times were compared using the log-rank test (**A**) Overall survival. Blue line, pN0 patients; 1184 deaths/1817 patients; 22.0 (20.5–23.5) months. Green line, pN1 patients; 2515 deaths/3182 patients; 15.0 (14.4–15.6) months. Grey line, patients without lymph node resection; 275 deaths/305 patients; 8.0 (6.9–9.1) months. (**B**) Cancer-specific survival. Blue line, pN0 patients; 1070 deaths/1817 patients; 24.0 (22.3–25.7) months. Green line, pN1 patients; 2342 deaths/3182 patients; 16.0 (15.4–16.6) months. Grey line, patients without lymph node resection; 254 deaths/305 patients; 9.0 (7.6–10.5) months.

More than 1 lymph node was examined in 4999 (94.2%) of the 5304 patients. The median number of lymph nodes examined was 12 (range, 0–72). There was no difference in TLN between patients with and without PORT (Table 1). The median overall survival durations for pN0 and pN1 patients were 22.0 (20.5–23.5) and 15.0 (14.4–15.6) months, respectively. Patients without lymph node resection had the worst overall survival duration at 8.0 (6.9–9.1) months (log-rank *P* <0.001). The analyses of cancer-specific survival had similar results (log-rank *P* < 0.001) (Figure 1).

### PORT and survival

Median overall survival times for patients who had undergone PORT were better than those for patients who had not undergone PORT (19.0 months; 95% CI, 18.2– 19.8 vs. 14.0 months; 95% CI, 13.3–14.7, respectively) (log-rank *P* < 0.001). The median cancer-specific survival durations for patients with and without PORT were 20.0 months (95% CI, 19.1–20.9) and 15.0 months (95% CI, 14.2–15.8), respectively (log-rank *P* < 0.001) (Table 1).

**Figure 2:**
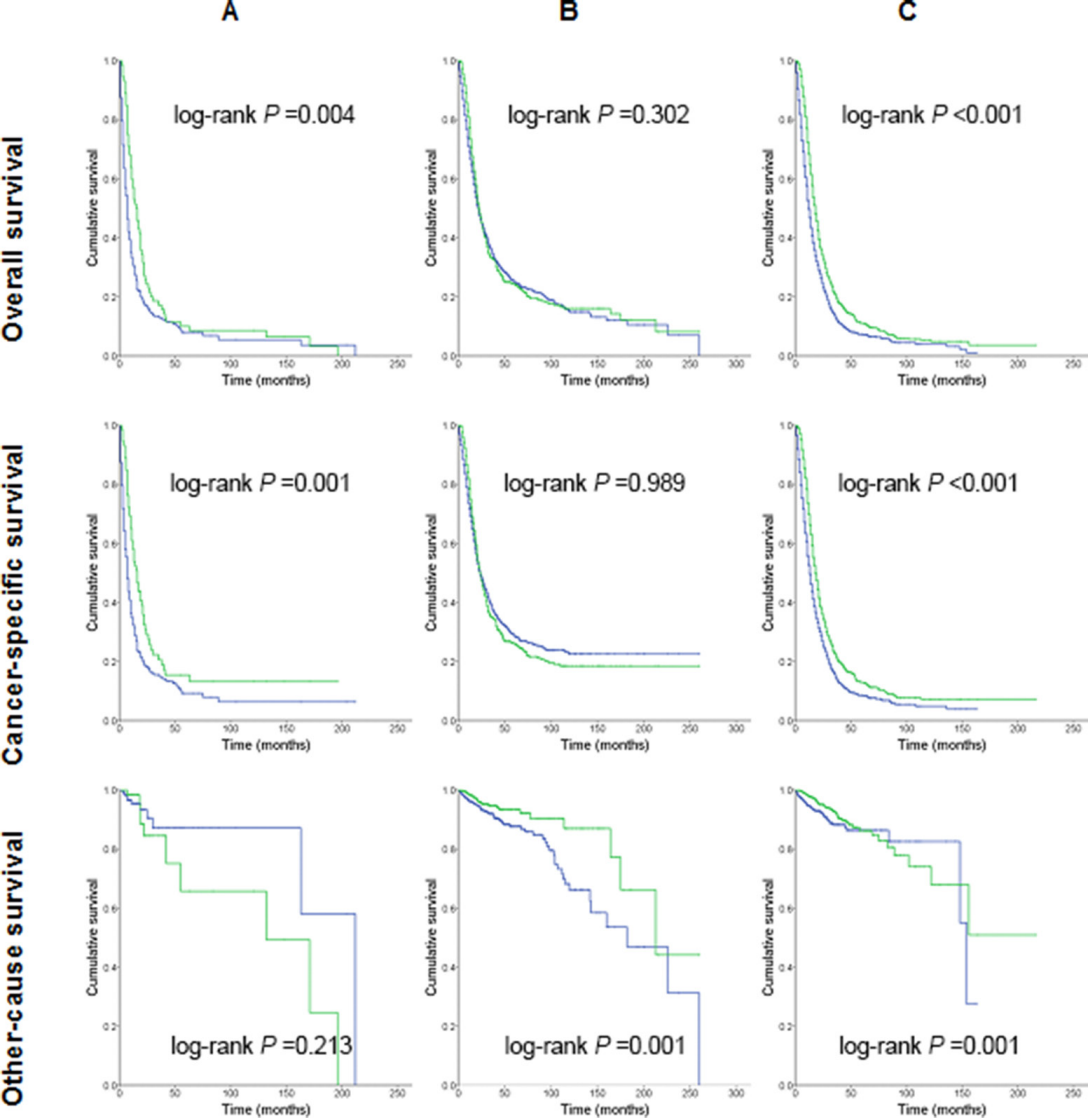
Kaplan-Meier survival curves for patients treated with or without postoperative radiation therapy Median survival times were compared using the log-rank test. Blue line, Patients who had not undergone postoperative radiation therapy; Green line, Patients who had undergone postoperative radiation therapy. (**A**) Patients without lymph node resection; (**B**) pN0 patients; (**C**) pN1 patients.

Figure 2 illustrates the survival curves for patients with and without PORT stratified by lymph node stage. PORT was associated with a significant increase in both overall and cancer-specific survival for the subsets of patients who had positive lymph nodes (N1 stage) and those who did not undergo lymph node resection. However, no such survival benefit was observed in patients with negative lymph nodes (N0 stage). Analyses also showed that patients with PORT had better other-cause survival than those without PORT.

Univariate and multivariate analyses based on the numbers positive lymph nodes and lymph node ratio in pN1 disease showed that patients who had undergone PORT had better overall and cancer-specific survival in each subset (PLN1–2, PLN ≥ 3, lymph node ratio (LNR) < 0.22, and LNR ≥ 0.22), but patients with pN0 disease did not (Table 2). In addition, there was a trend toward an overall and cancer-specific survival advantage in patients with LNR ≥ 0.22 who had undergone PORT.

**Table 2:** Survival analyses for mortality based on receipt of postoperative radiotherapy in patients with pancreatic adenocarcinoma

Clinical variables	No. of patients with postoperative radiation/total patients (No. of patients with missing values)	Univariate analyses, HR (95% CI)		Multivariate analyses, HR (95% CI)	
		Overall survival	Cancer-specific survival	Overall survival	Cancer-specific survival
**pN0**	699/1817 (48)	0.94 (0.84–1.06)	1.00 (0.89–1.13)	0.91 (0.81–1.02)	0.96 (0.85–1.08)
**pN1**					
** PLN**					
1–2	719/1654 (73)	0.68 (0.61–0.76)	0.69 (0.62–0.78)	0.67 (0.60–0.74)	0.67 (0.60–0.75)
≥ 3	601/1528 (58)	0.73 (0.65–0.81)	0.73 (0.65–0.83)	0.69 (0.61–0.78)	0.70 (0.62–0.79)
** LNR**					
< 0.22	702/1646 (72)	0.74 (0.66–0.83)	0.76 (0.68–0.86)	0.72 (0.64–0.81)	0.75 (0.66–0.84)
≥ 0.22	618/1536 (59)	0.65 (0.59–0.73)	0.65 (0.58–0.73)	0.62 (0.55–0.69)	0.62 (0.55–0.70)

Abbreviations: HR, hazard ratio; CI, confidence interval; PLN, total number of positive lymph nodes; LNR, lymph node ratio. Multivariable models adjusted for year of diagnosis (before and after year 2007) and tumor differentiation.

## DISCUSSION

Our study shows that PORT is associated with better overall and cancer-specific survival in pN1 patients with pancreatic adenocarcinoma. The benefit of PORT is maintained in stage N1 patients no matter how many positive lymph nodes are dissected. Patients who had undergone PORT had better other-cause survival than those who had not undergone PORT. This suggests that the addition of PORT not only extended the cancer-specific survival time by amelioration of the cancer itself but also decreased treatment-related death. Multivariate analysis revealed that the following factors were associated with shorter overall and cancer-specific survival: diagnosis before 2007, older age, black race/ethnicity, advanced-stage disease, tumor at body or tail of the pancreas, poorly differentiated or undifferentiated disease, LNR ≥ 0.22, and no PORT.

Several prospective trials have demonstrated that patients with pancreatic cancer benefit from PORT when it is included with chemotherapy. In Gastrointestinal Tumor Study Group trial 9173 (GITSG 9173), patients who underwent margin-negative resection and postoperative radiation with concurrent bolus 5-fluorouracil had a better median survival (20 months) than surgery alone (11 months) (*P* = 0.035) [7]. Other trials have shown no difference in median overall survival between patients receiving chemotherapy alone and patients receiving chemoradiation, although chemoradiation reduced the likelihood of local recurrence [8, 9]. Morak *et al.* also found no difference in overall survival for chemoradiation versus surgery alone but did find a progression-free survival benefit [10]. Moreover, patients with adjuvant chemoradiation had less pain, less nausea and vomiting, and improved global functioning [11]. Conversely, worsened outcomes with radiation therapy were reported by the ESPAC-1 trial (*n* = 289) regardless of whether patients received chemotherapy [12]. However, methodological flaws have prohibited an accurate evaluation of PORT in that study [13]. In a meta-analysis of 15 eligible randomized controlled trials including 1128 patients, chemoradiation could benefit the long-term survival of patients with locally advanced pancreatic cancer compared with chemotherapy or radiation therapy, although it may also increase treatment-related toxicities [14].

In addition, an analysis of SEER data between 1994 and 2003 showed a survival benefit for the use of neoadjuvant radiation therapy over surgery alone or surgery with adjuvant radiation therapy in treating pancreatic cancer. The median overall survival of patients receiving neoadjuvant radiation therapy was 23 months versus 12 months with no radiation therapy and 17 months with adjuvant radiation therapy. In multivariate analysis, they found a significantly lower hazard ratio (HR) for death in patients receiving neoadjuvant radiation therapy rather than adjuvant radiation therapy (HR 0.63; 95% CI 0.45–0.90; *P* = 0.03) [15]. In our study, the median overall survival was 19.0 months for those who received PORT versus 14.0 months for those who did not receive PORT between 1988 and 2012. The prognosis of pancreatic cancer is a very tough, though the survival rates have been improving particularly after 2007. The improvement may partially due to the early diagnosis and the development of treatment such as the introduction of gemcitabine as first-line treatment for patients with locally advanced adenocarcinoma of the pancreas. However, PORT remained as an independent good prognostic factor for pancreatic cancer patients with resection after adjusting for potential confounders such as year of diagnosis.

There are several limitations to this study. First, the analysis was retrospective in nature. As a result, treatment was not randomized. However, multiple imputation techniques showed similar results to the complete data imputation techniques, which indicates that, assuming the data are missing at random, the conclusions are robust. Secondly, the study data were affected by changes in the extent of resection and lymphadenectomy as well as in radiation techniques over the years. These changes were taken into account by adjusting for the year of diagnosis in the multivariable models. Finally, the SEER dataset does not contain information on pathologic margin status or adjuvant chemotherapy. The absence of information on chemotherapy may lead to an overestimation of the efficiency of radiation therapy when SEER data is used [16]. However, during the study period, adjuvant chemotherapy was recommended for all patients who underwent surgical resection and did not have significant contraindications.

Despite these limitations, our findings suggest that PORT is an important modality in the adjuvant management of patients with pancreatic adenocarcinoma. There is an immediate need for the evaluation of PORT in the treatment of postoperative patients with pancreatic cancer in prospective studies.

## MATERIALS AND METHODS

### Data source

We abstracted data from the National Cancer Institute's Surveillance, Epidemiology, and End Results (SEER) 18 registries database (November 2014 submission) [17]. In total, the database covers approximately 27.8% of the US population (based on the 2010 Census). Different years of diagnosis, ranging from 1973 to 2012, are included for different registries. The data reported in this study represent the most recent follow-up available in the SEER database (December 31, 2012).

### Cohort

We used SEER*Stat (version 8.2.1) to generate a case list. Patients aged 18–90 years who underwent primary pancreatic tumor surgery for which complete lymph node staging data were available, had a histologically confirmed first primary pancreatic adenocarcinoma, and were diagnosed between January 1, 1988, and December 31, 2012, were eligible to be included in the study. This SEER submission included cases through December 31, 2012, which would represent either the date of the last cancer diagnosis or the date of last follow-up. The exclusion criteria were survival time of less than 30 days after a confirmed diagnosis; a previous diagnosis of malignant disease; and pancreatic cancer reported from a nursing home, hospice, autopsy, or death certificate.

We generated a case list with information on the following variables: year of diagnosis, age at diagnosis, sex, race/ethnicity, marital status at diagnosis, tumor location, cancer stage, tumor grade, positive regional nodes number, examined regional nodes number, radiation, vital status, survival in months, cause-specific death classification, and other-cause-of-death classification. The last two variables indicate whether the person died of the cancer (cause-specific survival) or causes other than the cancer. TLN and PLN were also retrieved. LNR was defined as the PLN divided by the TLN. Because patients cannot be identified from the data, the Institutional Review Board of the First Affiliated Hospital of Soochow University exempted this study from review.

### Outcome measures

We used the vital status recode (study cutoff used), SEER cause-specific death classification, and SEER other-cause-of-death classification variables to extract data on the status of patients at the time of last follow-up. Based on this information, we calculated overall, cause-specific, and other-cause survival rates. We used the survival in months variable to extract information on time from the date of diagnosis to last follow-up. SEER*Stat estimates survival time in months by subtracting the date of diagnosis from the date of death or last contact. Cancer-specific survival was defined as the time from diagnosis to death from recurrent disease. We used the survival in months flag variable to identify missing or incomplete survival data.

### Statistical analysis

The patients’ clinicopathologic factors were compared using the χ^2^ and Mann-Whitney *U* tests. Survival times for different strata (ie. total and positive lymph node numbers) were compared using the log-rank test. We created Kaplan-Meier survival curves stratified by lymph node status. We performed a univariate Cox proportional hazards regression analysis to determine the hazard ratios (HRs) of death with respect to year of diagnosis (before or after year 2007), age, sex, race/ethnicity, cancer stage, tumor location, tumor differentiation, N stage, PLN (1–2 or ≥ 3), LNR (< 0.22 or ≥ 0.22), and PORT. We performed multivariable analyses on the association between PORT and survival duration, adjusting for all the applicable confounders listed above. All *P* values were two-tailed. A *P* value less than 0.05 was considered statistically significant. All statistical analyses were performed using SPSS version 22.0 (IBM Inc., Armonk, NY).
